# "The Hospital" Nursing Mirror

**Published:** 1901-05-04

**Authors:** 


*The Hospital*, May 4, 1901.

### "The Hospital" Nursing Mirror.

Being the Nursing Section of "The Hospital."

[Contributions for this Section of "The Hospital" should be addressed to the Editor, "The Hospital" Nursing Mirror, 28 & 29 Southampton Street Strand, London, W.C.]

### Notes on News from the Nursing World.

### THE ROYAL RED CROSS.

The King has conferred the decoration of the Royal Red Cross upon Miss A. Ward, matron, and Miss K. Neville, sister, in recognition of their services in tending the sick and wounded in connection with the recent operations in Ashanti. There has been comparatively little said about the work of the nurses attached to the West African Frontier Nurse, but it has been of a most valuable character, and the honour bestowed by His Majesty upon Miss Ward and Miss Neville will afford general satisfaction in the nursing world.

### THE PEKING NURSES.

Formal presentation has been made by the British Minister at Peking of the Royal Red Cross to Miss Saville and Miss Chapin for conspicuous services rendered by them in nursing the sick and wounded during the siege of the Legations. Miss Mary Lambert and Miss Jessie Ransome, of the Anglican Mission, will receive the decorations for similar services on their return to England. Miss Saville has been incorrectly described as a native of the United States. She is the daughter of an English clergyman, received her medical education at the London School of Medicine for Women, and holds the M.D. degree of Brussels. We cannot afford to make a present of any of our brave women to America.

### PLAGUE NURSING IN CAPETOWN.

We were able a fortnight ago to announce that a number of nurses had been engaged for plague work in South Africa at a salary of four guineas per week. The following left England a few days ago to take up duty: Misses Emily Blake, Louisa A. Boret, Alice Darly, Ada Charlotte Day, Victoria Evans, Margaret Gordon, Mary May Goy, Sarah Hancock, Katherine M. Hoddell, Alice Clara Holland, Eliza Johnstone, Maggie McCarthie, Ethel Macfie, Mary Middleton, Mary Nelson, Lucy Sarah Nottage, Margaret Palethorpe, Helen Payne, Lucy Sophia Price, and Ada Louisa Warman. Of these it may be mentioned that Miss Day has been second-class assistant nurse at the Fountain Fever Hospital, Lower Tooting, since August 1895. She was previously assistant nurse at Farnham Asylum, and charge nurse at the Metropolitan District Asylum, Caterham. Others have also had experience in the nursing of fever. For the information of the many correspondents who have written to us expressing a desire to volunteer for service we may state that all communications should be sent to Dr. Patrick Monson, Medical Adviser at the Colonial Office, S.W., who is acting for the Agent-General for Cape Colony; but at present no further nurses will be engaged. In the event of more being required, they would probably be wanted at exceedingly short notice. While there is no objection to applications being sent in to Dr. Monson, it may be well to warn nurses who think it worth while to offer themselves, that the prospects of being employed are remote, unless, of course, the epidemic should assume unexpected proportions. In addition to the matron of the Plague Hospital at Capetown, it is reported that two nurses who were inoculated with serum received from Bombay have died from the disease; but the Agent-General has not received any confirmation of the report.

### THE NURSES OF BELVIDERE HOSPITAL, GLASGOW.

Great enthusiasm has been excited at Glasgow by the fearless manner in which the nurses of Belvidere Hospital have discharged their duty during the course of the smallpox epidemic now, happily, believed to be at an end. The outbreak, which at first seemed likely to be of a temporary character, has been very serious, and the nursing has been as arduous as any in South Africa, without any halo of glory or element of adventure, save constant attendance on patients suffering from a most malignant malady. The actual danger of infection has been slight, but even a nurse may have a natural shrinking from coming in contact with smallpox. There is a suggestion that the nurses should be presented with some kind of decoration. This shows how anxious the people of Glasgow are that honour should be paid to whom honour is due; and whether decorated or not, the devoted staff at Belvidere Hospital have the satisfaction of knowing that their self-denying labours have been appreciated.

### MORE VAGUE CHARGES.

The speeches at the annual meeting of the Norwood Cottage Hospital, reported in another column, deserve a measure of attention because they are made by persons of some responsibility, and because a well-known member of the House of Commons threatens on certain conditions to bring the treatment of probationers before Parliament. But it will need evidence of a very different kind from that cited at Norwood to induce Parliament to take any action in the supposed interests of nurses. Why did the speakers confine themselves to vague assertions? If there have been cases of cruelty in any particular hospital, why are no names given? How can there be any investigation into statements which are as general as they are unfair? If a definite charge is made it can be dealt with; but even Mr. Tritton, M.P., who talks about "the terrible hardships" endured by a relative of his own at "a London hospital," refrains from indicating the institution. Meanwhile, the fact remains that no probationer enters a hospital blindfold. The preliminary month, or three months, which every candidate has to serve, is arranged as much for the protection of nurses as it is for the advantage of hospitals; and if wholesale cruelty is practised, why do probationers voluntarily expose themselves to it for three years?

### ROBBING THE NURSES OF ST. THOMAS'S HOSPITAL.

A daring, not to say audacious, robbery took place recently at St. Thomas's Hospital. The night nurses sleep in a building set apart for them, and during the day, when they occupy their rooms, they are locked into this detached portion. It was in the small hours of the morning, when the nurses were on duty in their several wards, that one of them, having obtained leave to go to her room to fetch something, saw a man slip out from another room at the end of the corridor. There was no way of escape for him except by passing her, and she was too scared, being alone and unprotected, to attempt to question or stop him. There was no one then in the building whom she could summon to her assistance. She, however, reported the occurrence as soon as possible, and it was found that several of the nurses' rooms had been entered, and watches, money, and jewellery extracted to a considerable amount. The beds had been in every instance disturbed, the pillows and bolsters being dragged about, as if the robber imagined that nurses were in the habit of leaving their watches under their pillows. The thief has not been discovered yet, but it is assumed that he must have been someone who knew his ground well.

### NORTHERN WORKHOUSE NURSING ASSOCIATION.

The tenth annual report of the Northern Workhouse Nursing Association is far from being a satisfactory document. The fears expressed by the committee the previous year were fully realised, and owing to inadequate support the work of the Association, especially the training of probationers, has been seriously curtailed. Instead of twenty, as in 1899, only thirteen were trained in 1900; but the deficit having risen from £175 to £250, the committee were compelled to make the reduction. They now appeal, it may be hoped not in vain, to friends of the Association to support it sufficiently to enable them to train the full former number. The staff at the present time consists of 96 members, of whom 17 are still in training, the remaining 76 comprising 18 superintendent nurses and 58 charge and assistant nurses, being divided amongst 45 unions. In view of the demand for nurses of the Association and the relatively limited number of the staff, no more than seven nurses are now supplied to any union. We agree with a paragraph in the report that "when the untrained though salaried nurse of the twentieth century has disappeared as entirely as the pauper nurses and 'Sairie Gamps' of the early years of the nineteenth, then only will the Association be no longer needed."

### CORK HOSPITAL FOR WOMEN AND CHILDREN.

It will be remembered that on March 2nd we announced the resignation of Miss Baxter, the lady superintendent of the County and City of Cork Hospital for Women and Children, and the entire nursing staff. We learn that there is no abatement of the strong feeling of indignation in Cork at the manner in which Miss Baxter has been treated, and her work overlooked by both the old and the new committee. A successor has, however, been appointed to her in the person of Miss Kathleen Disney, whose training and career leave nothing to be desired. But we are afraid that she has a formidable task before her. The annual meeting of subscribers was held on Tuesday. As, however, it was only advertised on Saturday the attendance was necessarily small. Miss Baxter's many friends in Cork are doing their best to keep her and her faithful staff in the city, and it is proposed to start a nursing institution.

### THE QUEEN VICTORIA MEMORIAL AT TUNBRIDGE WELLS.

No doubt there are many residents of Tunbridge Wells who have contributed, or will contribute, to the National Memorial to Queen Victoria. But this will not prevent an adequate fund from being raised in the town for the purpose of erecting the Nursing Institute, which it has been decided to build as a local memorial of the late beloved Sovereign. In addition to its appropriateness, the Institute will save the sum of £45 a year at least in rent alone, which, as it was pointed out at a meeting of ratepayers, will be of great benefit to the town. A statue of the Queen had been talked of, but the Tunbridge Wells people have very wisely selected a memorial of a character which will be of far more useful service than a monument of marble or stone.

### PROGRESS IN ROXBURGHSHIRE.

The proceedings at the annual meeting of the Roxburghshire Nursing Association were of a most satisfactory nature. To begin with, the balance to the credit of the working fund at the end of March was £104, or £30 more than at the corresponding period of last year, although the number of cases nursed was thirty-eight more than the number nursed in the previous twelve months. In view of the position of affairs, the subscribers were amply justified in proceeding to assent to the hire of a cottage as headquarters for four or five nurses in Melrose, and in increasing the honorarium of the secretary from £10 to £20. Another interesting feature of the gathering was a number of proposals by several parishes to be affiliated to the Association. It was decided to remit these applications to the consideration of the executive committee, but the fact that they should have been made indicates the growing appreciation of trained nursing north of the Tweed.

### "ECONOMY" AT RUTHIN.

Notwithstanding the statement of the Chairman of the Ruthin Board of Guardians that the Queen's Nurse has saved the Union many pounds during the past year, a majority of the Board have declined to continue the grant of £5 made in previous years. Mr. Bircham, one of the inspectors of the Local Government Board, was present at the meeting, and said he should be sorry to see the grant discontinued. He also observed that "any grant made to the Nurse's Fund would come out of the common fund of the Union, and anything saved by her services in Ruthin was a saving to the whole Union. But the assertion that "the services of the nurse were almost exclusively devoted to the town itself" was apparently considered of more importance than the arguments of the Chairman and Mr. Bircham. It may be hoped that there are few Boards of Guardians in the country who would be guilty of such ungracious and mistaken economy.

### AN EXAMPLE TO BOARDS OF GUARDIANS.

An interesting feature of the fourteenth annual report of the Ryde District Nursing Institute is the reference to an important concession on the part of the Guardians of the Isle of Wight Union, who during the past year gave permission to the nurses to call in the district medical officer to cases in their charge. Of course, the Guardians laid down conditions, but the concession has been of material value, as the committee of the Institute gladly acknowledge. It is always an advantage when the Guardians of a Union do their best to help, instead of to hinder, the labours of district nurses. In other respects, also, the Isle of Wight organisation is to be congratulated. The record of work done is admirable, the financial position is not discouraging, and it appears probable that the Nursing Home will shortly be enlarged and more nurses, who are badly wanted, employed.

### DUBLIN TRAINING TURNED TO ACCOUNT.

From Australia we hear that a sanatorium has been opened at Mitcham, a village 14 miles from Melbourne, in the heart of the gum forest, and yet within the suburban radius, by a nurse who was trained in the Irish capital. Mrs. Ballam came to the United Kingdom from Melbourne, and entered the Rotunda Hospital, Dublin, where she obtained her certificate. Returning to Australia, she established a sanatorium at Canterbury, a high-lying suburb of Melbourne, and the home she has now established is an auxiliary. It is pleasing to learn that the enterprise of Mrs. Ballam is appreciated at the Antipodes.

### ST. LUKE'S HOME, VANCOUVER.

From the report of St. Luke's Home, Vancouver, we learn that this excellent institution continues to be appreciated in British Columbia. In addition to Sister Frances, who is in charge, there are six nurses and two probationers on the staff, and they were kept going all through the province, being sent out to Ladner, Lulu Island, Eburne, Victoria, New Westminster, and the Similkameen country. Three of the nurses were presented with their certificates at a garden party, and another interesting event of the year was the marriage of one of the old nurses, who came back from up-country expressly to be married from the home. We are glad to see that, with the aid of donations from friends, the home has managed to pay its way.

### A START AT CREWKERNE.

At an influential meeting in the Victoria Hall, Crewkerne, it was decided to remove what the Chairman called rather a slur on the good name of the town, the slur being the non-existence of a parish or district nurse. Happily, in deciding to start one, the mistake of appointing a half-trained person which some people appear to have advocated was not committed. Nearly all the speakers, and especially the doctors, urged the importance of securing a fully competent nurse. Eventually, a committee, consisting of ladies and the medical men of the town as *ex-officio* members, was formed, and it was left for them to determine whether a nurse from the Queen Victoria Nursing Institution should be obtained. One of the speakers said he thought that there might be some little difficulty in raising the funds after the first year or two, when the novelty and interest in the movement had somewhat waned. But, apart from the fact that sufficient unto the day is the evil thereof, the experience of most towns is that when the work of a nurse is understood and its practical results can be appreciated, it is easier rather than harder to collect the necessary funds for her support.

### WEST BROMWICH NURSING ASSOCIATION.

We are informed that the report on which we founded some remarks about the work of the nurses of the West Bromwich Association was misleading. The honorary secretary of the House Committee assures us thatthe superintendent, as well as the two nurses, undertakes a full share of the number of cases attended; that each nurse has, in addition to a clear calendar month in the summer, a short holiday in the spring; that the nurses have never worked more than 8½ hours, generally not more than 8, daily, and that they have one Sunday in four off duty. Also that during the superintendent's holiday last year an extra nurse was engaged temporarily, and that in the winter months a probationer assisted the regular staff. It is a pity that these facts were not stated in the local papers, for they put the position in quite a different light. With regard to the new Nurses' Home, the site has been purchased by the committee at a cost of £300, and a sum of £1,000 has to be raised for the furnishing and endowment. But the building itself is to be the gift of Councillor Akrill. As the present temporary home has only accommodation for the superintendent and three nurses, more room is obviously wanted if, as the committee earnestly desire, the operations of the Association are to be extended.

### EAST-END MOTHERS' HOME.

The report of the resident lady superintendent of the East-End Mothers' Home, whose annual meeting will be held, by permission of Dr. Owen Lankester, at 5 Upper Wimpole Street, on the afternoon of Friday, May 10th, shows that the useful work has been carried on successfully and efficiently. The number of women admitted to the home was 248, and of children born 248; while the number of out-patients registered was 297. The home nurse, Miss Bentley, left at the end of December to enter a general hospital for further training, but a suitable successor has been obtained. With regard to the training school, out of fifteen midwifery pupils trained only three failed to obtain the certificate of the London Obstetrical Society. Thirty-three maternity pupils were also trained. The annual subscriptions amounted to £516 11s., and the donations, &c., to £1,057 7s. 1d.

### AN OLD-FASHIONED IDEA OF THE MODERN NURSE.

No. 30 was a very ignorant old man. His profession had been to travel round with a small circus and attend to the horses, and when first admitted to hospital he was one of the very dirtiest old "Daddies." The nurse talked to the old man sometimes; he rather approved of her, and said to one of her fellow probationers, "She oughter 'ave 'er pictur took, for a decent, hard-working young woman." One night some of the nurses had a theatre pass, and Daddy said to her, "What's the good of the likes of you and me going to the theatre? We shouldn't understand it!"

### A LEGAL PROTECTION FOR AMERICAN NURSES.

An excellent measure has been introduced in the American Senate, which contains a section requiring that in case of a trial in which a question may be put to a nurse touching upon information or knowledge obtained while acting professionally, she may be protected from answering it. The original and misleading announcement was that Mr. Slater was responsible for "a Bill forbidding trained nurses under the direction of a physician to disclose any information acquired from a patient during attendance in a professional character," a statement obviously suggesting that nurses chattered too much about matters which should be held sacred.

### SHORT ITEMS.

Sister A. Moatt, of the Indian Army Nursing Service, has been granted two months' sick leave, and transferred from Umballa to Dalhousie, on account of ill-health.—A café-chantant in aid of the District Nursing Home at Plaistow will be given on Tuesday, May 21st, at 24 Park Lane, thanks to the kind permission of Lord and Lady Brassey.—An appeal is made by the Dowager Countess of Longford for subscriptions in order to purchase comforts for the nurses in South Africa, which she is anxious to send out immediately. They should be addressed to Lady Katherine Pakenham, 24 Bruton Street, W.

### Notes of Surgical Lectures to Nurses.

Delivered at the London Temperance Hospital by Dr. W. J. COLLINS, M.S., B.Sc., D.P.H.Lond., Fellow of London University and of the Royal College of Surgeons. Surgeon to the London Temperance and the Royal Eye Hospitals.

### INTRODUCTORY.

THESE lectures are intended to deal chiefly with elementary anatomy, physiology, and pathology in their application to nursing. A medical man's training is arranged so that he shall first study the normal and then the abnormal or diseased body, and treatment or therapeutics is reserved to the last. A probationer, on the other hand, usually has to do with treatment from the first and acquires her knowledge of disease, and even in many cases her first knowledge of elementary anatomy and physiology, while engaged in the wards in carrying out therapeutic instructions. This arrangement does not facilitate the education of the nurse. To meet this anomaly it is convenient to proceed in each lecture, as far as possible, from the normal to the abnormal, from the simple to the complex, from the surface to the deeper structures, from physiology through pathology to treatment. Practical instruction is supplied in the wards by the matron and sisters, and every nurse would do well to read Florence Nightingale's "Notes on Nursing," and the article from the same eloquent author in "Quain's Dictionary of Medicine." Access to text-books on anatomy, physiology (such as Gray's, or Quain's and Kirke's), and anatomical plates, or, better, a visit to the Hunterian Museum at the College of Surgeons will be of great assistance.

It should be unnecessary to emphasise at the outset the importance of training the power of observation. To observe accurately and report faithfully constitute a large portion of the equipment of an efficient nurse. It is difficult to lay down precisely the extent and depth of anatomical and physiological knowledge essential for good nursing. High schools and Board schools both affect to teach something of both. M. Foster's "Primer of Physiology" will give a general notion of the subject to those who have had no previous instruction of the kind, but a nurse will do well to refer from time to time to a systematic work on any point or case on which she feels the need of fuller information. This habit of reference should be encouraged and facilitated.

Sir Charles Bell was in the habit of illustrating his lectures on anatomy by demonstrations on a living subject, in order to show how much of the structure of the body can be learnt without actual dissection, and it is well to practice marking with coloured pencils the bony land-marks felt through the skin, to trace the lines of the main blood-vessels and nerves, and locate with precision the regions occupied by the various viscera. The X-rays have helped as never before to define with exactitude the relation of the bones to the contour of the surface. To an anatomical eye or to surgical fingers the deeper parts should stand revealed with similar precision as the result of inspection and palpation.

A general acquaintance with the human skeleton is assumed. The upper extremity supplies examples of the three chief varieties of bones, the flat, *e.g.* the scapula; the long, *e.g.* the humerus; the short, *e.g.* the carpus. Any other kind of bone is spoken of as irregular or mixed. If a section through the middle of the upper arm is looked at, there are seen from within outwards (1) skin; (2) a layer of fat; (3) fleshy masses of muscle separated by layers of fascia; (4) blood-vessels running in the long axis of the limb and chiefly collected in a sheath between the bone and the inner side of the limb (a slight examination will serve to show the vessels are of two kinds, the thicker walled arteries, and the thinner and more numerous veins); (5) nerves running alongside the vessels though not in the same compartments of fascia as the vessels, and distinguished from the latter by being white fibrous cords not tubes; (6) a more minute search may reveal yet another kind of vessel, viz. the lymphatic, not easily identified in health, but in some forms of blood poisoning greatly engorged and readily traced by red lines on the surface; (7) the bone—the humerus with its outer layer of compact ivory-like tissue, surrounded by periosteum, its inner layer of cancellous tissue and its cylindrical cavity full of marrow or medulla.

Bone, chemically, consists of organic matter (gelatin) $\frac{1}{3}$ and inorganic matter (salts, chiefly calcium phosphate) $\frac{2}{3}$. In the disease known as rickets the proportions may be reversed and the bones may bend and bulge. Bones are developed from cartilage or membrane by ossification or deposition of earthy salts; the same process which confirms and consolidates a bone after it has been broken. Some bones ossify from one central spot, whence the salts spread to the outlying surfaces; other bones, especially long bones like the humerus or radius, ossify from more than one centre. In the radius of a boy of twelve three pieces can easily be distinguished, viz., the shaft and the two ends or "epiphyses;" the two latter do not, owing to their having been developed from separate centres of ossification, unite with the shaft in one continuous piece until at or after the age of puberty. Before this date a separation of one or other epiphysis is apt to be mistaken for a fracture.

A fracture has been grandiloquently described for more than two hundred years as "the solution of continuity of a bone," but is not usually held to include separation of epiphysis. The cause of fracture is force applied directly or indirectly from without, or by muscular action from within. A broken leg from a cart-wheel running over it is an example of direct external violence; a broken collar-bone from a fall on the outstretched arm is a case of indirect external violence; and a fracture of the patella from a slip on a piece of orange peel is an instance of fracture due to muscular action from within. There are also contributory or predisposing causes of fracture, *e.g.*, age, sex, bone degeneration, malignant or constitutional disease, rickets, &c.

Fracture is "simple" (unconnected with wound of skin) or "compound" (connected with wound of skin), single multiple or comminuted, complete or incomplete (greenstick) impacted or not. The signs of fracture are deformity crepitation, abnormal mobility and maiming. Most fractures, if the broken ends are accurately coapted (set) and, kept at rest, unite by bone, formed from and by the periosteum and adjacent bone, after the blood has been absorbed and the ends ensheathed in "callus." In some cases there is non-union, and in others, *e.g.*, patella, neck of femur, &c., the union is commonly effected by fibrous tissue. Repair takes six to nine weeks and is usually slower in compound than in simple fractures. Bones of the arm are more often fractured than bones of the leg. The radius is the bone most frequently broken and especially its lower end. The lower end of the upper fragment is not unfrequently driven into the upper end of the lower fragment; that is to say it is "impacted." Thus, crepitation, which is elicited by the rubbing together of fractured surfaces is absent, but there may be considerable deformity owing to non-reduction, and this may result in vicious union.

Bones are brought into relation with other bones or

"articulated" by means of joints, which may be movable like the knee or immovable as the sutures of the skull. Movable joints are hinged (knee, elbow), ball and socket (hip), rotatory (as the radius around the ulna) or gliding (like the vertebral articulations). The articular ends of bones are smooth by virtue of being covered with cartilage; these again are lubricated with synovial fluid secreted by a synovial membrane which invests the fibrous or ligamentous capsule holding the bones together.

As the result of accident or disease the normal relations of the articular ends of bones may be disturbed and a "dislocation" results. The shoulder is the most frequent joint to undergo dislocation, generally from a fall on the outstretched hand; dislocation of the hip is a common occurrence in the later stage of hip disease, due to softening of the ligaments and dislocation of the head of the femur, resulting in shortening of the leg.

In a muscular subject many of the muscles of the arm and the tendons or fibrous leaders in which they terminate can be easily identified beneath the skin. If the arm is raised to a right angle with the trunk the front of the arm-pit or axilla is formed by the graceful outline of the pectoralis major muscle whose chief action is to fold the arms across the chest, and in birds it is the great muscle of flight. This broad triangular muscle is attached at one end to the clavicle, the sternum, and the cartilages of the ribs, and at the other to a ridge two inches long on the front of the shaft of the humerus. The attachment to the humerus is spoken of as the "insertion" of the muscle, and the other broader attachment is spoken of as the "origin" of the muscle, in accordance with the rule of anatomists who designate the point on which a muscle acts or the more mobile of its attachments as the "insertion" and the point or points from which it takes its pull, usually the less mobile of its attachments, as the place or places whence it "arises" or its "origin."

### Queen Victoria's Nurses.

THE promoters of the meeting at Londonderry House on Monday afternoon have every reason to congratulate themselves so far on success, for the large room was well filled by a representative and influential audience. Blue uniforms were scattered up and down the room, and among other representatives of the nursing world were Miss Peter, Miss Guthrie Wright, and Miss Rosalind Paget.

### Lady Londonderry's Speech.

The chair was taken by the Marchioness of Londonderry, whose opening speech was as follows:—

"In rising to inaugurate our proceedings to-day, I would thank you on behalf of myself and the other ladies whose names are attached to the letter published in the public press on April 15th last—Miss Florence Nightingale, the Duchess of Buccleuch, the Duchess of Portland, the Marchioness of Londonderry, Countess Cadogan, Countess of Lonsdale, Countess of Strafford, Countess of Selborne, Lady Penrhyn, Lady Blythswood, Lady Lucy Hicks-Beach, Lady Victoria Lambton, Lady Ponsonby, Mrs. Hertz, Mrs. Kenrick, Mrs. Power Lalor—for the hearty manner in which you have responded to our appeal, and for your presence here to-day, in order to consider the best mode of promoting a Women's Memorial to the late Queen. If you will allow me, I will recall to you one sentence in the letter, and that is: 'We feel very strongly that there could be no more fitting Memorial raised by the Women of the British Empire to Her late Majesty, our Beloved Queen, than to collect a sum of money to carry on the work which Her Majesty herself started, *i.e.*, to provide the poor with good nurses in their own homes.' Our reasons for thinking so are as follows:—Many in this room will recollect the time when it was not considered at all necessary to procure trained attendants for sick people. It was during the time of the Crimean War that a revolution in the manner in which hospital patients were nursed was wrought by the pioneer of scientific and trained nursing—Miss Florence Nightingale—this being one of the great social changes for the better which have taken place during the late Queen's long and glorious reign. It is not so long ago that there was great difficulty in getting experienced skilled attendants for those to whom money was no object; and now, when it is comparatively easy to do so, we wish to make it possible, so far as lies in our power, for the poorer members of our community to obtain trained nurses. We hope to achieve this end by endowing the Queen's Jubilee Institute for Nurses, which institution trains nurses for district work and assists with grants districts which are too poor to help themselves. We know very well many may say that the statue that is to be erected in London will be the national memorial to Her Majesty Queen Victoria. We fully acknowledge this, and recognise that it will be a fitting record of the glory and grandeur of her reign; but in asking you here to-day to help us to organise the women's memorial we wish to perpetuate the remembrance of the deep sympathy with sorrow and suffering which Her Majesty invariably extended to all her subjects; for in showing this sympathy Her Majesty not only proved that she was a great and noble Queen, but that she was also the loving mother of her people."

### Mr. Sydney Holland's Appeal.

An eloquent appeal was then made by the Hon. Sydney Holland, who said he had been asked, or rather commanded, to speak on the subject because he was at the head of the largest training school for nurses in London, and also because he was on the Queen's Commemoration Council. "They wanted," he said, "to endow this great work, so that they would no longer have to go from pillar to post begging for means to continue Queen Victoria's work, and fulfil the sacred trust she left. The Queen Victoria Jubilee Institute (he hated the word Institute) was merely two rooms up at St. Katharine's, from which the whole of this gigantic work was carried on. The cost of each nurse was £60 a year. The Institute had to inspect the nurses scattered all over the United Kingdom, and help with money grants those districts which were too poor to help themselves. He had received many touching excuses from ladies to whom he had written asking them to help. One had bidden him pray always, but she had spelt it 'prey.' He would gladly do either the one or the other, or both. Another had said she was already obliged to give up her biscuits at lunch on account of the calls upon her purse. He was very sorry for her. This was an appeal from women to women. Forty-five years ago Florence Nightingale sacrificed her health for the cause of nursing, and now she had come forward again to ask the women of England to continue this work. In her message to them she had said, 'God speed your noble effort,' and her blessing should have very great weight. It was almost a platitude to say it was the best work in which a woman could engage. The Queen's Nurses taught unselfishness, kindliness of heart, and the rules of health and hygiene, and, most important of all, they made things very much better for the children. Many children but for the nurse would die of neglect. Was the audience aware that if there were 100 people in that room, all of them blind, 30 would be blind because of the ignorance of the mother at birth? It was so; 30 per cent. of the cases of blindness in children were from this cause. A nurse had lately boasted that she had taught all the mothers of little children in her country district to walk slower, because one of the commonest injuries from which little children suffered was dislocation of the shoulder from being dragged along the street and pulled up by the arm when they fell. They were able to lessen the number of burnt children too. As most people knew the signs of spring, hospital authorities knew the signs of winter: the cots filled up with burnt children. One branch of the work, which a good many of the Queen's Nurses took up, was the attendance at confinement cases. Could those present realise what it was at such a time to have no comforts, no extra warmth, no invalid appliances, a door and window that would not shut, and the bed probably occupied till the last moment by other children? There was another point. People sometimes said, 'Why do you want a nurse when there are the hospitals?' But hospitals could not be everywhere; they would be useless in scattered districts; and in a crowded district they were so overwhelmed with appeals to be taken into the wards that, practically, the answer had daily to be, though not in those words, 'You are not near enough to death. When you are nearer death we will take you in.' He did not think there was any advantage in a child being nursed in the hospital if it could possibly be nursed at home. Surely, they could not allow such a work as this, started by the Queen, carried on by the Queen, help for which was asked by Florence Nightingale, to be starved for want of money."

### The Resolutions.

The following resolution was then proposed by the Duchess of Portland and seconded by the Duchess of Roxburgh:—

"That this meeting cordially approves of the movement, initiated by the Committee of the 'Queen's Commemoration Fund on behalf of Queen Victoria's Jubilee Institute for Nurses,' to raise an Endowment Fund in memory of her late Majesty Queen Victoria, and pledges itself to make every effort to secure the success of the appeal to the women of the United Kingdom on behalf of the Queen's Nurses Endowment Fund as a fitting tribute to the memory of Queen Victoria, who herself started the scheme for nursing the sick poor in their own homes with a sum of £70,000 from the Women's Jubilee offering of 1887."

The next resolution was proposed by the Marchioness of Lansdowne and seconded by Alice Countess of Strafford:—

"That for the working of the appeal in England and Wales the following ladies be asked to form a Committee, with power to add to their number:—Duchess of Beaufort, Duchess of Marlborough, Duchess of Bedford, Duchess of Portland, Duchess of Westminster, Marchioness of Lansdowne, Marchioness of Londonderry, Dowager Countess of Shrewsbury, Countess of Derby, Helen Countess of Radnor, Countess of Lonsdale, Countess of Selborne, Countess Roberts, Lady Sandhurst, Lady Hilda Brodrick, Aileen Lady Belmont, Baroness Burdett-Coutts, the Lady Mayoress, and Mrs. Beerbohm Tree; that the arrangements for Scotland be left in the hands of the Scottish Council; and that the Countess Cadogan be requested to undertake the formation of the Committee for Ireland."

### A Practical Suggestion.

Mr. Harold Boulton, Hon. Sec., proposed a very warm vote of thanks to Lady Londonderry for calling the meeting. He would invite them to make their vote of thanks a real one, such as Lady Londonderry would wish. If they asked themselves, "How much do I sympathise?" they might find a method of measuring their sympathy on certain slips of paper placed about the room. But there was more they might do. If any, having heard of the meeting at Londonderry House and being pleased that the acquiescence of those in the highest quarters had been obtained, should add, "Now let us go away and try to divert it to our local association," to these people he hoped they would point out that that would not be "fair cricket." They might be assured that there were and always would be plenty of people with a particular genius for raising funds for local objects. Local patriotism was always alive. It was this central national organisation that required putting upon a sound financial basis. The benefits of inspection from headquarters reached the local organisation, and a rich district that could afford to be self-supporting should have the satisfaction of knowing that it was helping one that was too poor to help itself. He begged them to take a large national view of the matter, not a narrow parochial one, and thus fitly to perpetuate Queen Victoria's memory.

### Royal British Nurses' Association.

### General Council Meeting.

The quarterly meeting of the General Council of the Royal British Nurses' Association was held on Friday, at the offices, 10 Orchard Street, Mr. Pickering Pick in the chair. After the reading of the minutes of the last meeting, Mr. Langton, Hon Treasurer, read the financial report, in which it was shown that £519 15s. 10d. had been received during the quarter, and £525 0s. 3d. expended. There was a balance at the bank of £96 18s. 7d. The accounts for this quarter compared very favourably with the corresponding quarter of 1900; the Settlement Account was also to be congratulated on the amount it had received. Before asking the meeting to adopt the statement, which had already been passed by the executive committee, he would make a few remarks about the *Nurses' Journal*. Whereas hitherto it had been a loss to the Association, it had now been able to contribute to the funds. The report, after being duly seconded, was adopted, and Mr. Fardon then read the report of the Executive Committee. Forty-two nurses had been registered, 38 had been elected members, three had resigned their membership, and one death had been reported.

The Committee had resolved to defer the issue of the roll of members for this year and for the future until after the annual meeting of the Corporation and succeeding council meeting had been held, in order that the names of members of the General Council and Executive Committee then elected might be printed in the roll. It was hoped that the roll of members containing the names of new members to June inclusive might be ready by the end of July, and the Committee believed that it would be found very convenient for reference to have a correct list (not immediately superseded, as had hitherto been the case) of the Council and Committee. It was intended to issue the roll at the reduced cost of 1s.

Sessional lectures of a very interesting character had been delivered during the past winter. Mrs. Humphry Ward gave an address on Special Classes for Invalid Children; Mrs. Tennant lectured on The Work of the Industrial Law Committee; Dr. Hill, of Pankhoi, lectured on Medical Mission Work in China, and the final lecture was by Mr. Richard Greene, on Ancient Medicine and Ancient Medical Men. It had been a great advantage to be able to hold the lectures at the office of the Association, and members appeared to have appreciated the preceding reunions.

The annual meeting of the Corporation had been fixed to take place at the Imperial Institute, on Thursday, June 6th, at 3 p.m.

Mr. Fardon said it was proposed to make some arrangement for the nurses to go to the Crystal Palace after the annual meeting. It was the Jubilee year of that institution; there would be an exhibition and a musical entertainment, and, moreover, Thursday was one of the celebrated evenings for the display of fireworks. He thought that members, especially those from the country, would enjoy it, and it was hoped that the arrangement would be carried out on advantageous terms.

The report, having been seconded, was adopted. Miss Scott proposed that a vote of thanks be sent to those ladies and gentlemen who had kindly lectured for the Association during the winter session; the attendance had been exceptionally good, and the little social tea which preceded them seemed to be greatly appreciated. Mrs. Coster seconded, and a vote of thanks to the chairman for presiding, terminated the proceedings.

### Examination Questions for Nurses.

### PREPARATIONS FOR OPERATIONS IN PRIVATE HOUSES.

### Result of April Competition.

The question was as follows:—"If called to an operation, such as abdominal section at short notice in a small house, what preparations should you make in the room, and with regard to the patient?"

### The First Prize.

"Nurse Eileen" is the successful candidate for the first prize. The answers this month have reached a higher standard, and the question appears to have been popular, for an unusual number of answers have been sent in. The only fault to be found with "Nurse Eileen's" is, that in all probability she would not have time to remove carpet and scrub the floor. In the event of only having a few hours it is unwise to remove a carpet, although its presence is so objectionable, because the dust raised is still more pernicious. In such a case I should leave the carpet alone and ask for old sheets, tacking them firmly down so as to cover the floor and prevent dust rising from the passage of feet.

### The Second Prize.

"Ivanhoe" wins the second prize. Her paper is excellent and practical, but alas! there is "a little rift within the lute." She does not mention that both the hot and cold water must be sterilised. No doubt she is aware of this, but in competition nothing can be taken for granted. But for this omission she would have come out first.

### Honourable Mention.

"Bobs," "Nurse Molly," and "Nurse Dar" have gained honourable mention, and there have been many other papers of almost equal merit.

### First Prize.

I should endeavour first to set my patient's fears at rest, keeping her as quiet as possible. With this end in view, I should endeavour to prepare a room for the operation in close proximity to the patient's bedroom on the same floor; then remove the carpet and all unnecessary articles of furniture, having the the floor well cleaned. For an operating table, the kitchen table may have to answer the purpose, and should be placed as near the window as possible. Two small tables would doubtless be procurable, which, covered with white cloths, would hold instruments and lotion bowls; basins are easily obtainable, and a pail will be needed. There should be a fire burning in the room, and an abundant supply of boiling water, and also cold sterilised water. Have a fish kettle filled with water in which the instruments may be boiled. For spray sheets, a few yards of white American cloth would answer the purpose; also three or four towels should be sterilised, and laid in water pending the surgeon's arrival.

I should give the patient an enema of soap and water (1 pint), and also see that the bladder is emptied immediately before the operation; wash the body all over, and give as much liquid nourishment as time would permit, such as egg and milk, milk, or beef tea; half a pint of the latter being given just four hours before the operation. The abdomen might be shaved, rubbed with a little turpentine or methylated spirits, and a compress of sterilised water applied, if the part is not too tender. If the patient is not able to take nourishment owing to persistent vomiting I should give sips of hot water, and if thirsty allow the mouth to be rinsed out frequently with water, mixed with a little lemon juice if preferred. The hair if long should be made into two plaits. An iron bedstead with a spring mattress is the best, but a straw one may have to be used; the bed should be made up clean to receive the patient, have a bolster at hand to place under the knees, two bricks or small wooden boxes will answer the purpose of blocks, and a stool with the legs shortened might be used as a temporary cradle.

### Second Prize.

I should have a fire lighted; clear operating room of all unnecessary articles of furniture; procure a strong table, if one not long enough, two ordinary dressing tables of equal height would do; place in best possible light; over table spread several folds of blanket, on this a macintosh, and over all a folded cloth to serve as draw sheet, not forgetting to set a small pillow at the top to support patient's head. Convenient to hand of anæsthetist, place a basin and several small cloths in readiness in case of sickness during operation. If procurable I should have another table, if not, a wash-hand stand, or top of chest of drawers would do, cover with a clean cloth; on this I should place basins for sponges, lotion, &c., also surgical instruments, dressings, &c., and a little brandy in case of need. Have in readiness a basin or some kind of empty vessel to receive whatever may be removed from patient, also hand-basin, soap, towels, plenty of hot and cold water, and bucket, or some kind of receptacle for waste. An old table or crumb-cloth placed under operating table will serve to protect the floor.

I should remove all hair in vicinity of proposed opening. Empty bladder and rectum as short a time as possible before operation, wash all over, if time allow, being particularly careful to thoroughly clean that part of abdomen where incision is likely to be made. If possible to obtain, patient should have on a wool vest, wide enough to admit of being turned up, a warm flannel nightgown, and long wool stockings. As vitality is greatly lowered under influence of anæsthetic, patient should be kept as warm as possible, and no part unnecessarily exposed. All hair-pins should be removed and hair neatly braided in two plaits. When placed on the operating table, patient's head should be slightly raised; night dress, &c., quite open at the throat. A small blanket, folded in two, should be placed crosswise over upper part of chest and abdomen, and a larger one, similarly folded, across lower portion and extremities.

When patient is pronounced quite ready, raise night-dress and vest well above abdomen; fold in upper blanket to leave sufficient space for operation; have a clean towel wrung out of carbolic lotion and place on chest, over blanket, turning end well in, so that carbolic be in immediate contact with the skin. The edge of lower blanket might be turned down in like manner, a mackintosh placed over it, and over this, again, a carbolic cloth as before.

In meantime prepare bed as comfortably as you can for the patient's reception; have clean linen, if possible, and a hot-water bottle for the feet; instead of upper sheet place blanket next patient.

A stout straw pillow to place under the knees will greatly relieve abdominal tension.

### Pseudonyms.

Such names as "The Kid" and "Wire-haired Terrier" are inadmissible.

### The Question for June.

In a case of fractured thigh treated with a long splint, in what places should you watch for splint sores, and what means should you take to prevent them?

The Examiner.

### Rules.

The competition is open to all. Answers must not exceed 500 words, and be written on one side of the paper only. The pseudonym, as well as the proper name and address, must be written on the *same* paper, and *not on a separate sheet*. Papers may be sent in for fifteen days only from the day of the publication of the question. All illustrations strictly prohibited. Failure to comply with these rules will disqualify the candidate for competition. Prizes will be awarded for the two best answers. Papers to be sent to "The Editor," with "Examination" written on the left-hand corner of the envelope.

N.B.—The decision of the examiners is final, and no correspondence on the subject can be entertained.

In addition to two prizes honourable mention cards will be awarded to those who have sent in exceptionally good papers.

### "The Hospital" Convalescent Fund.

The Hon. Sec. begs to acknowledge with many thanks the receipt of £1 from Mrs. Bridges and of 1s. 4d. from Miss Sophie C. Borgaes.

### The Co-operation Awakens.

ONLY three weeks ago we felt it our duty to call the attention of the nurses of The Nurses' Co-operation to the mismanagement of their affairs. Our warning came not a day too soon, as subsequent events have proved. There are 500 nurses on The Nurses' Co-operation, and more than 400 of these have already sent in a protest against the high-handed proceedings of the committee, which culminated in the resignation of Miss Hughes, the lady superintendent, and the immediate election of Miss Wells, the matron of the Howard de Walden Home, to succeed her. There are at least 50 nurses abroad out of the minority who have not yet protested, and therefore it may be seen that the whole of the nurses are now alive to the manner in which their interests are being imperilled by the two or three women who manage the committee.

### A MEETING OF THE NURSES.

We understand that a general meeting of the members will shortly be called, but this does not mean a meeting of the nurses, as the members number only about twenty-five. Now it is most desirable that the nurses should meet and should take a definite stand with regard to their Co-operation. The dissatisfaction of many years has come to a head, and it remains with the nurses to have sufficient courage to voice their opinions and desires. The question must be faced by the nurses, "What is to be done in order to regain the control of our business?" In regard to the present management, it must be remembered that the members of the committee had nothing whatever to do with the founding or original organisation of the Co-operation, and that since their reign the affairs of the Co-operation have sunk into a very parlous state. On money grounds alone it is a very serious consideration that over £10,000 of the nurses' savings is sunk in a Home which does not pay its way. This makes the financial responsibility of the committee in regard to the use they have made of the nurses' money a very serious one. It must be considered whether the proper course would not be for the present nurses to withdraw, and under Miss Hughes to form a new Co-operation, leaving the legal members of the present institution to proceed against the Committee of Management in order to recover the savings of the nurses which have been sunk in the Home. But, if the committee have any self-respect, they will promptly resign after being condemned by the whole body of the nurses. At any rate, within the next week or two steps will be taken either within the Co-operation or, if the Committee of Management prove obdurate, without the Co-operation, to call a meeting of all the nurses who can possibly attend. It is of the utmost importance that a strong sense of public duty and a high professional spirit should be displayed at this crisis. Let the Co-operation remember the great work which it did at the beginning. It was the first successful union of nurses to secure their own interests and to maintain the status of their profession. Many nurses in those early days gave time and money, and bore abuse and calumny in order to start this great movement. Unfortunately, as so often happens in prosperity, sloth for a time fell upon the Co-operation, and it was in this interval that the present handful of members took it upon themselves to rule this large union of women.

### THE NURSES' REPRESENTATIVES.

Nurses will do well to study the Articles of Association, and to consider whether they have been treated as they ought to have been treated by their representatives. We would particularly call attention to Section G of Article 30 on page 15 of the Memorandum and Articles of Association, dealing with the powers of the Committee of Management, and also to Section B of Article 34 on the same page, dealing with the disqualification of members of the Committee of Management. They read as follows:—

Article 30, G. "They shall formulate and circulate among the nurses of the Society such regulations as they may think fit for enabling such nurses from time to time to hold meetings for the election of their representatives on the Committee of Management, under the provisions in that behalf hereinbefore contained. Any such regulations which shall conform as nearly as possible to the regulations of the Society for the convening and holding of General Meetings shall prescribe the mode and extent of any notice to be given of any such meetings, and the rights and mode of voting thereat, and, generally, shall provide for all arrangements and regulations in relation to the holding of and proceedings at such meetings."

Article 34, B. "The office of Member of the Committee of Management shall be vacated if he cease to be a member; or, being a representative of the nurses, if he does not strictly conform to the regulations formulated by the Committee of Management under Article 31, Section G.

We are informed that regulations have not been circulated amongst the nurses such as enable them to hold meetings and to formally elect their representatives on the Committee of Management, and, when the whole body of nurses rises up against the decision of the Committee of Management, it is perfectly clear that the term "nurses' representatives" as applied to those who are at present on the committee is a mere farce. These people do not represent the opinion of the nurses, and by hook or by crook the nurses have got to regain the control of the Co-operation.

### THE CO-OPERATION MUST SPEAK.

We understand that Miss Wells has refused to take up the appointment vacated by Miss Hughes, but all this is a mere matter of gossip, and the nurses who form the Co-operation seem to be the last people to hear anything about what is being done. If it is a fact, however, it shows that there is some sense of loyalty yet remaining amongst the officers of The Nurses' Co-operation.

And now that at last the public and professional spirit is awake once more, it is not only for themselves, but for the nurses all over England, nay, all over the world, that the Co-operation has got to speak. If any body of persons consents to be tyrannised over and enslaved, they wrong not only their profession but also their whole sex. Therefore it is that we ask the Co-operation nurses to come forward with spirit and confidence, and, for the sake of themselves and others, to support those who have the real welfare of their union at heart.

The position of the doctors and surgeons who have helped in the formation of and done such yeoman service in the cause of the Co-operation is a very peculiar one. We cannot but hope and believe that those who have stood by the nursing profession so staunchly in the past will be with the nurses in the future. We are certain they would be the last to wish their names to be made public as joined with those who have done much to spoil one of the best organisations for the most hardworking class of women. Indeed, it will be a serious matter for all those whose names have to be made public as being connected with what is little short of a scandal with regard to a great public trust.

If any nurse who is abroad or who has not yet received any notice with regard to the protest which has been signed will communicate with us, we will put her in touch with those nurses of the Co-operation who have had the courage to come forward and lead at a time when, but for their efforts, the whole Co-operation might have been wrecked.

### Metropolitan Nursing Association.

ANNUAL MEETING AT GROSVENOR HOUSE.

By the kindness of the Duke of Westminster, the Metropolitan Nursing Association again held their annual meeting at Grosvenor House, on Monday afternoon. The chair was taken by Mr. H. Bonham Carter. There were also on the platform Sir Dyce Duckworth, M.D., Mr. F. D. Mocatta, the Rev. Dacre Craven, and Mr. W. Minet. The Chairman alluded to the loss sustained by the Association in the death of the late Duke of Westminster. No meeting was held last year, so that they really had to consider two years' work. The late Duke had been very closely connected with the Association—formerly called Metropolitan and National. He was the first Chairman, and had continued in that capacity till the end, ever ready to assist with valuable advice and practical help. He then called upon Sir Dyce Duckworth to propose the adoption of the report.

Sir Dyce Duckworth hoped all present had read the report carefully through, and that they would agree that the year's work had been in every way satisfactory. The Association had been carrying out their objects in a most satisfactory manner. He was not sure that he was historically accurate about the relationship between this Association and the Q.V.J.I., but he had always believed that this Association was the mother, and the Q.V.J.I. the child. As one of the council, he could testify to the splendid piece of work carried on by the Q.V.J.I. in the kingdom. In the past poor people had suffered exceedingly if unable to go to the hospitals, but now things were very different. In order to produce nurses of the required qualifications much training had to be gone through—the minimum was two years in hospital and six months in the district. Both were essential; *esprit de corps* was always necessary, and this came through life at the nursing home. Some people were ambitious enough to desire a three years' hospital course; at present it seemed rather too ambitious. Our friends north of the Tweed claimed to be able to compress into two years' training that which took us three years to accomplish. Referring again to the Queen Victoria's Jubilee Institute, he said it was progressing by leaps and bounds. Scotland had gone ahead, England had gone ahead, and Ireland was going ahead. For these reasons the Association must commend itself to their judgment.

Mr. F. D. Mocatta, in seconding the adoption of the report, said that of all the societies in London he knew of none which more thoroughly merited support. He was glad to see Mr. and Mrs. Dacre Craven in the room: they were connected with the Association from the beginning. At that time people were much influenced by the writings of Charles Dickens, and they felt that the nurses of those days were most incompetent to fulfil their duties. Great progress had been made since then, and progress was still being made. We had had the testimony of our late beloved Queen to the value of nurses: she placed their services very high. From Bloomsbury Square a number of similar institutions had sprung, both in London and the provinces; this was a great blessing. The nurse was now hardly second to the doctor, for the doctor had to pass on to another patient, while the nurse carried out his orders. The nurse left her influence behind her when the case was ended; the patient recollected how she opened the windows, dusted the room, &c., things which formerly were considered not only unnecessary, but injurious. He was very glad that the Association had met again in that room, and he hoped that what was said there might spread beyond its limits.

The report having been adopted, Mr. W. Minet proposed a resolution pledging the meeting to support the Association.

Dr. Hawkes said that the last time he had the privilege of speaking at one of these meetings he had been able to say from experience what the nurse's work was in the slums. Now his work took him more among the classes between those who could pay a guinea or two for a private nurse, and the very poor, and he was convinced that there was as great a need among such for skilled nursing as among the poorest; in fact, the very poor were better off in that respect. The nurse's work was not easy, and the time that it might be imagined was devoted to rest was frequently spent in attending or preparing for lectures, which were very necessary. One of the great values of the work was that it was educational: only those who had worked among the poor had any idea of their ignorance and superstition. The owners of "open surgeries" had been known to complain that since the nurse came the patients got well too quickly. He was often exasperated by the ignorance of the poor; but when he remembered that well-to-do people had spent £4,000 in one year on a palmist it was not surprising. Miss Hughes had urged the attendance of the nurses at confinement cases, and he heartily supported her; but he believed the committee was opposed. This was, in his opinion, very foolish, and he thought that if they could see the drunken old creature that still existed they would agree with him. "There are times now," said the speaker, "when I just pray that I had my old friends the Bloomsbury nurses to help me." He approved of a nurse being a nurse only, and not trying to combine other things with it. He had himself been nursed by a Queen's Nurse, and could speak from experience of her value.

The Rev. Dacre Craven then gave some financial details regarding the house in Bloomsbury Square, and the alterations carried out during the year on the piece of ground available at the back; the £1,200 they were going to borrow from the bank would be well laid out. Some of the nurses had been sleeping in another house for some few years, and he wished to testify to the manner in which they had put up with the inconvenience, which had in no way hindered their work. The committee was grateful to all the nurses, and especially to Miss Haddon, for their work, and the loyal way in which they supported the committee; £50 a year was to be put by to repay the loan, which would thus take twenty-four years to pay off. He would like to see it repaid in twenty-four months.

Mr. Bonham Carter then asked Miss Twining to come upon the platform and say a few words, and she complied. She said she was the oldest member of the Association present. She was of opinion that more might be done by ladies in the country; she had started, or helped to start, four associations when living in different places—at Kensington, Worthing, Tunbridge Wells—and an industrial centre near Rochester. She thought more should be done also to spread the knowledge of the nurses' work among the tradespeople. In all the branches with which she had to do the nurses left slips of paper suggesting that those should pay who could afford it. There was too much free medical advice.

Mr. Bonham Carter quite concurred as to payment being made by those who could afford it, but said the tradespeople must have their own nurses. He did not see why there should not be a corps of nurses in every parish; but to supply the tradespeople was far beyond the scope of the Queen's Nurses in every way.

The meeting concluded with a vote of thanks to the Duke of Westminster.

### To and From the Front.

A CHAT WITH A RESERVE SISTER.

A NUMBER of nursing sisters from South Africa have arrived at Southampton on board the s.s. *Nubia*, *Aurania*, *Orotava*, and *Orient*. The following is the official roll:—Sisters A. Guthrie, L. Basan, L. Dale, B. J. Betty, and M. Atkinson did duty on board the *Nubia*, while Sisters J. G. Lambe and E. E. Lloyd were invalided home. On the *Aurania* were Sisters A. W. Walkinshaw, Gertrude Fletcher (both from the Imperial Yeomanry Hospital, Deelfontein), F. M. A. Coates, and Eva Whistler (A.S.N.R.), and Sisters Susie Bell and E. L. Phillips (Civilian Nurses). The *Orotava* had a long roll, as follows:—Superintending Sister M. C. Fisher (Imperial Yeomanry Hospital, Deelfontein), B. Lancaster, A. E. Lee, A. Timbrell, G. Tillett, G. Rogers, M. L. Loveday, H. Rooke, F. B. Lambert, E. C. Macpherson; and Sisters Hill and Babb were invalided home. The s.s. *Orient* brought Sisters Scott and Show (Ladysmith and Howick), time-expired, and Sisters A. Williams, B. Williams, and Sister Rogers on sick leave.

NURSING ON THE VOYAGE.

"We were all pleased and astonished," said Miss Walkinshaw, "at the clever way in which the ship was turned into a hospital: there were really two hospitals, or what, in a house, you would call two different floors, one each end of the ship. There were swinging cots for the most serious cases, and we had a good many of these. Two patients died, and we landed twenty-two stretcher-cases. There was a suicide on board, too, which was very painful."

"Was there much illness?"

"Yes, a good deal. Of the two who died, one developed enteric on the fourth day after leaving; he died two days before reaching Southampton. It was terribly sad, for he was an only son, and such a nice boy. The other was a case of dysentery."

"I hope you had a comfortable voyage."

"Very, except for the heat in the tropics—where we were very glad of the electric fan—and the rough weather after leaving St. Vincent. Everyone was so kind and attentive on board, and the invalid diet was splendid, I believe."

"Are you returning to the Cape?"

"I don't know yet, but I am quite ready to go if the War Office wishes to send me."

"The experience has been useful, has it not?"

"I think so. Terrible as the war has been, and still is, it has given us experience that we could have gained in no other way."

A LITTLE VISITOR FROM THE TRANSVAAL.

"Did any Boer prisoners come to the hospital?"

"Yes, there were two. One, funnily enough, was a boy called Christian De Wet. He was wounded—by accident, I think—and not in open warfare. The name must be a favourite one, for I know of a Dutch parson of the same name. This boy was very amusing, and I used to tease him sometimes by putting this little creature on his bed; he was terrified at her."

The little creature in question was sitting, while we talked, quietly on Miss Walkinshaw's lap: it was a rock marten, and had a most fascinating foxy little face, with bright brown eyes and large sensitive ears. The telephone bell was ringing frantically at this moment, and "Baby" was all nerves. She feeds on mice in her wild state, and has sharp, burrowing claws, which have grown long during her voyage. "I do hope she will not die of cold," said her owner; "I have got so attached to her; she was brought by a patient from the Transvaal, and is such an affectionate little thing. She barks at the dogs; and the other day she went for a huge mastiff. She doesn't follow, even in leash, and I only dare let her loose in my room; I am so afraid she will run away. To-night I am taking her to friends; she doesn't understand English soil, it's not good to burrow in." "Baby" was a great joy at Deelfontein, where the isolation must have been rather terrible.

IN CAMP.

"The nearest farm was three and a half miles away," said Miss Walkinshaw, "occupied, of course, by Boers. But we were so well agreed among ourselves that the time was very pleasant. There was a good library, which, however, I don't think we made very much use of, for being off duty meant only being away for an hour or so, with plenty of odds and ends for oneself to be done."

"What is the Karoo like?"

"Just a bare plain with kopjes; the camp was under a kopje. It was such a refreshment when we came down, to see some green trees and grass: everything was so burnt up on the Karoo."

A YEAR ON THE KAROO.

"Did you enjoy the life at Deelfontein?"

"Yes, it was a very happy time. We were particularly fortunate in not losing any of the sisters. Doctors and orderlies run a great risk, and we have lost from their ranks, but the sisters' risk is really greater, for they are so constantly touching the patients. Like so many others, I had an attack of enteritis, and as I had friends about 300 miles away I went to them for a few days, instead of resting in camp."

"One would think twice before travelling 300 miles at home, for so short a time!"

"Yes, but all distances are so great out there that one doesn't think of them in the same way. For instance, we were 500 miles from Cape Town, yet we did not think much about going down there. We were on the line to De Aar, which is 29 miles north of Deelfontein."

WHY THE NURSING STAFF CAME HOME.

"You had forty sisters at first, had you not?"

"Yes, when we had 1,000 beds. But the number of beds has been reduced latterly to about half, and there were about thirty sisters when we left. The hospital has now been taken over by the R.A.M.C. as Number Twenty-one General Hospital, and this is why the staff has been changed. Our orderlies were civilians: some belonged to St. John Ambulance. They did their work admirably, and were so anxious to learn. Some of the sisters have gone elsewhere in the Colony, and the rest came home. Miss Fisher was coming with us, but she found it difficult to get away so soon, so she followed a week later in the *Orotava*, and has just arrived."

"Were you hospital nursing before going out?"

"I was private nursing, and had only recently joined the Nurses' Co-operation. I was trained at St. Bartholomew's."

### To Nurses.

**WE invite contributions from any of our readers, and shall be glad to pay for "Notes on News from the Nursing World," or for articles describing nursing experiences, or dealing with any nursing question from an original point of view. The minimum payment for contributions is 5s., but we welcome interesting contributions of a column, or a page, in length. It may be added that notices of entertainments, presentations, and deaths are not paid for, but, of course, we are always glad to receive them. All rejected manuscripts are returned in due course, and all payments for manuscripts used are made as early as possible after the beginning of each quarter.**

### Echoes from the Outside World.

### AN OPEN LETTER TO A HOSPITAL NURSE.

It may interest you to know that there was a sale at Tattersall's last week of a large number of the horses which belonged to Queen Victoria, and with which she was accustomed to drive at Windsor. The sixteen animals brought in a total of 1,421 guineas. The King, of course, has no further need of them, as he already possesses a number of his own, and also probably does not require such steady, quiet horses as naturally found favour in the eyes of an old lady troubled a good deal with lameness. But I am so glad to see that he has refused to part with the two white donkeys which were always used to draw the little chaise about the grounds of the Queen's various residences. Wherever she went of late years—the South of France, Ireland, Scotland, or the Isle of Wight—the familiar chaise and the familiar donkeys went too; and it is pleasant to read that, now that their work is over, the faithful animals are not to be disposed of, but by the King's order have been placed in one of the Royal paddocks at Hampton Court.

---

The latest production at the Savoy Theatre has an extraordinary interest for everyone, because it is the last opera from the pen of the late Sir Arthur Sullivan. There are altogether twenty-eight musical numbers; of these Sir Arthur completed two only. But he wrote the melodies of fourteen more, and although Mr. Edward German has had to harmonise and orchestrate them, the public will love to remember that the airs at least of the principal part of "The Emerald Isle" were written by the musician who for so many years provided for them hours of pleasure at the familiar Savoy. But few who listen to the new opera can fail to feel grateful to Mr. German for the masterly and loving way in which he has surmounted the difficulties of finishing the work for the stage; and the twelve numbers which he himself supplies are very full of merit, and make one hopeful in thinking of Sir Arthur's successor. The play is very Irish, very whimsical, very well mounted, and very well acted. The Lord Lieutenant of Ireland and his wife (Mr. Jones Hewson and Miss Rosina Brandram) always speak in blank verse and walk about in their State robes. His daughter (Miss Isabel Jay) acts prettily as the damsel beloved of the young rebel (Mr. Robert Evett) who, contrary to preconceived ideas of Irish rebels, has been to Eton and Oxford, and is sadly ashamed of the loss, in consequence, of his Irish accent! Miss Louie Pounds is a charming peasant girl, to whom a fiddler, who has pretended all his life to be blind, is devoted, and a great deal of the fun of the piece is supplied by Mr. Walter Passmore, who dances, sings, and acts in his usual inimitable manner as "a professor of elocution, mesmerist, humourist, character impersonator, and general illusionist" summoned to Ireland by the Lord Lieutenant to teach elocution in the infant school. The libretto is by Captain Basil Hood, and it is sometimes exceedingly "Gilbertian." Decidedly nurses who want a treat and a good laugh should not fail to find their way speedily to the house on the Embankment.

---

Again the Guildhall is open free to the public and those who love pictures will be as anxious to see this tenth display as they were to inspect the other nine which have preceded it. This year the show is devoted to the productions of Spain, and the canvases include 91 by famous painters of the past and 117 examples by more modern men. Amongst those who have kindly lent pictures are King Edward, the German Emperor, the Queen Regent of Spain, and patrons of art in Italy, France, and the United States. The collection, though very representative and full of the best in Spanish art, is not too comprehensive to be enjoyed in an afternoon off duty, and the nurses who may find their way into the City are likely to specially notice "The Seat of Philip II.," a large canvas by Alvarez; "Don Balthazar Carlos in the Riding School," by Velasquez; "The Spanish Marriage," and the "Head of a Negro of Morocco," by Fortunys, and "Dr. Payrel," by Goya. The latter is only a three-quarter portrait of a man in a pale grey coat, with a blue-patterned white waistcoat, but it is a wonderful picture, and should not be missed.

---

The first week in May is devoted to what Mr. Robert Newman calls "The London Musical Festival." The name is a little misleading, as there is no choir-singing, but the performances given are all excellent of their kind, and those who love to compare the different methods of different great men, have had ample opportunity, for the concerts, alternately evening and afternoon, have been conducted by five different conductors—M. Colonne, M. Ysaye, Dr. Saint-Saëns, Herr Weingartner, and Mr. Henry J. Wood. On Monday M. Colonne took the lead, and, strangely enough, he succeeded in getting from the ordinary Queen's Hall orchestra a far finer performance, both in the "Tristan" prelude and in the "Venusberg" music from "Tannhauser," than he did from his own band the last time he was over in England. Signor Busoni played the solo part of Liszt's pianoforte concerto in E flat with marvellous power and brilliancy, but wisely refused to do more than acknowledge the plaudits of an audience who thundered for an encore. For the benefit of those who had no opportunity of being present on Monday, I may mention that great musical treats are promised on the afternoons of May 23rd, June 6th, 13th, and 20th, when Ysaye and Busoni will be the soloists, and that three afternoons in June will be devoted respectively to Wagner, Beethoven, and Tschaikowsky.

---

I suppose that as long as this wicked world lasts there will be men—and women too, sometimes, though they are not the worst offenders—who will try to secure their own ends by bribery and corruption. A case has just been reported in the papers wherein a butcher carrying on business in the north of London made an unsuccessful attempt to bribe a servant of the Metropolitan Asylums Board in connection with tenders for meat, and was fined by the Recorder £500. An instance, which unfortunately is less likely to receive punishment, has lately come under my own notice, and is specially interesting because it shows the temptations to which servants are exposed, and which one is proud to know they frequently surmount. A young cook, upon going to the door one morning to take in the usual supply of milk, found that the can had disappeared with its contents, evidently stolen. So she ran to the nearest dairy to buy what was required for breakfast. The man in charge, recognising that she was a new customer, inquired who usually supplied the household, and if her mistress were satisfied. The girl replied in the affirmative, and added that the same milkman had supplied the family for seven years, though she herself had only been in their service a few weeks. "Then," continued the tempter, "it is quite easy for you to earn a little money. Just water the milk every morning, and the lady will soon complain. It is quite easy, no one will find out, and the day you bring the custom here there will be 5s. for you. I have given away £2 or £3 that way lately." But for once he addressed the wrong girl, for the maid went straight back and told her mistress.

### Everybody's Opinion.

[Correspondence on all subjects is invited, but we cannot in any way be responsible for the opinions expressed by our correspondents. No communication can be entertained if the name and address of the correspondent is not given, as a guarantee of good faith but not necessarily for publication, or unless one side of the paper only is written on.]

### THE NURSES' CO-OPERATION.

"MISS HONNOR MORTEN" writes: As a member of the above co-operation I wish to protest against the action of the committee in accepting the resignation of Miss Hughes without consulting the Society, and in filling up the vacancy privately. Surely such an important post ought to have been advertised, and filled by fair competition. This further example of the autocracy of the committee has, I rejoice to see, aroused the nurses from their sleep.

"ROME" writes: Ought not the nurses of the Co-operation to be informed why their matron has resigned and another has been appointed without their being made aware of the facts? I always understood Miss Hughes was most popular with the nurses, whose interest she had at heart, and efficient in discharging her duties; also the friends of my patients have constantly commented to me upon her charming manner, good business capacity and judgment, when they have had occasion to call at the office. Is it not the duty of the representatives of the nurses on the committee to have some means of finding out what are the wishes of the nurses as a body upon matters of business which immediately affect them? They are practically the supporters of the Co-operation, and surely ought to have a voice in the management of the same. I would suggest that the nurse representatives should call a meeting of all the nurses who are within reach of London to talk the matter over, and come to some general understanding.

"A GRIEVED 'CO.' NURSE" writes: That the nurses on the staff of the Nurses' Co-operation have taken too little interest in the management of their society, there can be little doubt. "Aprons" truly writes: "We are so continuously busy with our cases." Nurse representatives are nominated or re-elected every year. We are asked to vote for them or others, and here we have let our affairs rest. But we are growing older, and realise the need of more co-operation amongst the members, and we also want a larger number of nurse representatives on our committee. We are grateful to a committee which has done so much for us, but the feeling is growing that matters are cramped, and some airing is needed. The acceptance of Miss Hughes's resignation and speedy appointment of a successor—three days after the resignation was accepted—has aroused indignation and suspicion. Miss Hughes can never know how much she is worth to us, nor how highly we value her keen high-minded judgment and general goodness. Over 400 nurses have petitioned that Miss Hughes's resignation be not accepted, and this together with such fine feelings of love, loyalty, and confidence, which were at the same time expressed, and which we know have always existed, by such an overwhelming majority of the nurses, is sufficient evidence from their point of view. The votes of all the nurses could not be taken, as there are at least 50 "Co." nurses working in South Africa and abroad. Therefore, any cause for her resignation which we can remove, we must make a stand for, and do all in our power to make it possible for Miss Hughes, if she will, to remain our matron. For the lady who is said to have been appointed Miss Hughes's successor one can only feel sorry, as her position must be extremely awkward.

### THE CROYDON CRISIS.

"FAIR TREATMENT" writes: Doubtless "Justice" and "Deeply Interested" are ladies of Miss Julian's type, and it is one word for Miss Julian and two for themselves. I knew Miss Julian personally and consider her unsuitable for the position of matron. I was nurse at Croydon and so know what I am speaking about. Certain nurses I know required weeding out, but surely a matron who knows her work can find that out in less than three years. I know that after the first year's service Miss Julian told a nurse she was not suitable. Surely in less than a year it could be found out if a nurse were suitable. Of course there must be discipline. I should think any nurse would understand the rule of "do as you would be done by," but on the other hand I think matrons ought to have a little tact. I am not asking for a favoured judgment but fair treatment. I think outsiders cannot possibly understand the matter, and should leave the Croydon Guardians to manage this affair themselves.

### IN REPLY TO A FRENCH WITNESS.

"ERALC" writes: I read with much regret the letter of a French witness. Personally, I do not believe the statements. Anyhow, if they are true it is but fair to hear the testimony of one who has found that nuns as nurses are self-sacrificing and entirely above all mock-modesty. Some years ago I nursed a case with a French sister (Bon Sécour order). She did night duty and I day. Our patient was a very severe case of Bright's disease. Any nurse knows how trying such cases become, especially towards the end, and how many unpleasant duties the nurse has to perform. I was young to nursing in those days, and I felt keenly many times about the nature of the work. But sister's unselfishness, devotion to her duty, and untiring patience with my grumbling always made me ashamed of myself, and helped me to rise above all petty feelings. I have many times helped her to give the patient a blanket bath, and she not only knew how to do it, but did it thoroughly. Indeed, all her work was done to perfection, and I many times realised what a difference there was between our work. Mine being entirely for £ s. d. lacked much of the thoroughness and refined finish of hers, which was done from higher motives. I am much the better woman as well as the better nurse from that nine weeks' association with the "unsexed" or "hypersexed" nun-nurse.

"A LOVER OF JUSTICE" writes: "A French Witness" say in your issue of 27th inst. that he or she considers nuns to be hypersexed somebodies, denying their self-sacrificing and devoted lives. That amongst nuns, as amongst every other organised body, there are to be found both good and bad is undeniable, but your correspondent seems to have seen some strange cases of mock-modesty. Permit me to say that for the past eight years I have been in frequent contact with nuns of various orders, both at home and abroad, and I cannot speak too highly of their devotion and increasing watchful care and attention. Some years ago I was a frequent visitor at the Hospital of St. John of Jerusalem, Great Ormond Street; and also the Italian Hospital, Queen Square, served respectively by Sisters of Mercy and Sisters of Charity; and the patients there, male and female, Catholic and non-Catholic, were unanimous in their praise of the attention and skill of all the nuns. I myself was taken ill with a severe attack of appendicitis some three and a half years ago in Oporto, whilst staying in a visitation convent, and found them as willing as an ordinary English nurse to do all that both the Portuguese and English doctor required, albeit in the presence of either or both these men, never forcing me or requiring me to see them alone. As to the riches spoken of pertaining to the well-known Societies of the Little Sisters of the Poor and St. Vincent de Paul, this is a fallacy. The good they do, the privations and hardships they endure, make them beloved as much by Protestants as by the members of their own faith.

### "SHE WOULD SAY 'SIR.'"

"SISTER GRAY" writes: In reading the article on "Ethics of Nursing" on April 20th I was somewhat surprised at the sentence, "No well-trained nurse would address the doctor either by his name or as 'Doctor.' She would say 'Sir.'" Is not this a rather sweeping assertion? I should be glad to know if it is the general opinion of the nursing world. I was trained in a small provincial hospital in which it was the rule for sisters and nurses to address physicians as "Doctor" and surgeons as "Mr. So-and-so." I am aware that in large London (and many provincial) hospitals it is a rule for nurses to address the medical men as "Sir," and think it would be absurd indeed of a nurse to refuse to comply with any rule of an institution which she had voluntarily entered; but surely under other circumstances the use of the term may be left to the discretion of the nurse. I cannot see that what is necessary and fitting in a large institution is necessary and fitting in private work, under such very different conditions. Surely it is possible to address a doctor by his name or merely as "Doctor" in a perfectly suitable manner without being either flippant or nonchalant. I am convinced that many of your readers who can deservedly be called "very well-trained nurses" have not been taught that this particular point is essential. Is not this a very insignificant test by which to judge of the excellence of a nurse's training?

NURSING TYPHOID UNDER DIFFICULTIES.

"A NURSE" writes: Last autumn I was asked to nurse an outbreak of typhoid in a borough in the South of England. I did not care very much about the work, as I was down south convalescing myself from an attack of typhoid. However, I undertook it, and found on reaching the place that the room procured for me was more than half a mile from my patients. I had to walk through mud and rain, do my visits, returning to lunch at 1 P.M.; going back to my patients and doing my night visit, which extended far into the night very often, whilst sometimes I had to stay all night. One of my patients, a young girl of sixteen, developed phlebitis. A very officious neighbour called one afternoon in my absence and propped up another with pillows, the result being an attack of syncope. The other four made an uninterrupted recovery; but one poor woman was very ill for a long time. During the time I was nursing I had to wash linen, scrub floors, and do all sorts of work; but the good recoveries my patients made repaid me for all my trouble.

### Norwood Cottage Hospital.

### SUGGESTED LEGISLATION FOR PROBATIONERS.

AT the annual meeting of the life governors, donors, and subscribers of the Norwood Cottage Hospital on Saturday evening, after the report had been adopted, Dr. GALTON moved that the best thanks of this meeting be given to the matron and nursing staff for their continual kindness and attention to patients. He acknowleged that some six years ago he owed his life to devoted skilful nursing. He was only too glad of any opportunity in public or private life of expressing his appreciation of the services which matrons and nurses rendered either in the wards of hospitals or anywhere else. Dr Galton then referred to the way in which he alleged that probationers at some large hospitals were maltreated. "These poor young girls, with no one to speak for them, were kept on their feet for very long hours, some being actually engaged for fourteen hours a day. Parliament had done a service to shop girls, and yet here were ladies, to whom perhaps they would owe their lives, only allowed two hours recreation. He had known the daughter of a clergyman in that neighbourhood who was poisoned by the bad food in a hospital. Some public man with a reputation like their president should bring this matter before Parliament. These poor girls in the hospital had to wash out their lockers and so on, which was all right, but many were required to use 20 per cent. solution of carbolic acid. Would any of those present use it? It was cruel, brutal, and unnecessary. The management of the hospitals, so far as the treatment of probationers was concerned, needed public attention being attracted to it."

Dr. GANDY seconded the resolution. He could endorse everything said by Dr. Galton with regard to the question of the education of nurses. "It had," he said, "come within his ken to have received complaints from nurses as to their treatment in London hospitals. He did not know any more noble act that the president or any other person occupying a similar position could undertake than that of ventilating the hospital treatment of nurses."

Mr. C. E. BLUNDELL said at one of the hospitals he could name the nurses were very badly treated. "They were compelled to go up three, four, and five flights of stairs, whereas the students of the hospital were allowed to use the lifts. They were, in fact, over-driven, like a cab horse or a dog out coursing at its last gasp. Yet they were ladies. It was a cruel thing to expose ladies to undertake such work."

The PRESIDENT (Mr. Tritton, M.P.) said from his own knowledge he was perfectly satisfied that the statement made was no exaggerated one. "He had a relative who had been working some time in a London hospital, and Mrs. Tritton would bear him out in saying that her health was completely broken down under the strain. He thought that the terrible hardships to which these young ladies were exposed was a matter that ought to be looked into. He did not know if it was exactly ready for Parliamentary interference. If Dr. Galton would submit questions to him, and he (Mr. Tritton) thought they were questions which might be put in the House of Commons, he should be very pleased to put them. They had a way in the House of Commons of calling attention to such matters as these by putting questions to a responsible Minister."

The meeting soon afterwards terminated.

### Appointments.

BRADFORD CHILDREN'S HOSPITAL.—Miss Lily Barraclough has been appointed staff nurse. She was trained at the Gateshead Children's Hospital.

CHESTERFIELD AND NORTH DERBYSHIRE HOSPITAL.—Miss Edith A. Hurst has been appointed male ward and theatre sister. She was trained at the Royal Infirmary, Preston.

CORK HOSPITAL FOR WOMEN AND CHILDREN.—Miss Kathleen Disney has been appointed lady superintendent. She was trained at the London Hospital, where she was afterwards night superintendent. Subsequently she was matron at Boston Hospital, and matron superintendent at the Royal Infirmary, Preston.

HASTINGS UNION INFIRMARY.—Miss Elizabeth Pitt has been appointed assistant nurse. She was trained by the Meath Workhouse Nursing Association at St. Joseph's Hospital, Chiswick.

HORNSEY ISOLATION HOSPITAL.—Miss Alice Giles has been appointed matron. She was trained at Guy's Hospital for three years, and afterwards held the post of sister at the Royal Portsmouth Hospital and the General Hospital, Birmingham, successively. For the last six years she has been night superintendent at the London Fever Hospital.

LISKEARD COTTAGE HOSPITAL.—Mrs. Winifred Butler has been appointed matron nurse. She was trained at the Queen's Hospital, Birmingham, and has since been ward sister in the same institution.

REIGATE ISOLATION HOSPITAL.—Miss I. M. Dundas has been appointed matron. She was trained at the Glasgow Royal Infirmary, and has since been matron at the Glasgow Ophthalmic and matron at Leith Fever Hospital.

### Presentations.

PRESTON ROYAL INFIRMARY.—Miss Edith A. Hurst, who has just completed her three years' training at Preston Royal Infirmary, and is leaving to take up an appointment at Chesterfield, was on Monday evening presented with a very pretty pair of pictures from the sisters and nurses. The matron was present at the musical entertainment which was also given in Miss Hurst's honour.

**Erratum.**—In our issue of the 27th ult. the article describing the Military Hospital, Valetta, a correspondent points out it should have been the Civil Hospital, Valetta.

### For Reading to the Sick.

"TAKE UP THY CROSS."

WHAT is it Jesus saith unto the soul?
"Take up the Cross, and come and follow Me."
One word He saith to all men: none may be
Without a cross yet hope to touch the goal.
Then heave it bravely up, and brace thy whole
Body to bear; it will not weigh on thee
Past strength; or if it crush thee to thy knee
Take heart of grace, for grace shall be thy dole.

Give thanks to-day, and let to-morrow take
Heed to itself; to-day imports thee more,
To-morrow may not dawn like yesterday:
Until that unknown morrow go thy way,
Suffer and work and strive for Jesus' sake:—
Who tells thee what to-morrow keeps in store?
*C. Rossetti.*

God sends the Cross of weakness and powerlessness to teach us that there is but one thing in the world worth seeking, and that is, to execute the Divine Will. The important point is not to be strong, learned, active—what really concerns a man is, to be in his place in the eternal monument which God is building; and if our place is one of suffering, impotence, premature death—Amen.—*Perreyve.*

To live for others, to suffer for others, is the inevitable condition of our being. To accept the condition gladly, is to find it crowned with its own joy.—*Westcott.*

Do not let us wait to be just or pitiful or demonstrative towards those we love until they or we are struck down by illness or threatened with death. Life is short, and we have never too much time for gladdening the hearts of those who are travelling the dark journey with us. Oh! be swift to love, make haste to be kind!—*Amiel.*

In order to be satisfied even with the best people, we need to be content with little, and to bear a great deal. Even the most perfect people have many imperfections, and we ourselves have no fewer. Our faults combined with theirs make mutual toleration a difficult matter, but we can only "fulfil the law of Christ" by "bearing one another's burdens." There must be a mutual, loving forbearance.—*Fénélon.*

Constancy and faithfulness mean something else besides doing what is easiest and pleasantest to ourselves. They mean renouncing whatever is opposed to the reliance others have in us.—*Anon.*

And ever, as we rest within the shadow
Of Thy long waiting time,
Our waiting days grow better, holier, grander,
Their service more sublime.

Until at last we hear Thy dear Voice saying,
"Child, I have need of thee
To fill this vacant place of trust and honour,
To do this work for Me."
"Then think not thou art kept within the shadow
Of long inactive years,
Without some purpose infinitely glorious,
Some harvest sown in tears."

And so there comes a glory and a gladness
Into the weary days,
And in our hearts there shines a solemn radiance,
Inwrought with quiet praise.—*Anon.*

### Notes and Queries.

**The Editor is always willing to answer in this column, without any fee, all reasonable questions, as soon as possible.**
**But the following rules must be carefully observed:—**
**1. Every communication must be accompanied by the name and address of the writer.**
**2. The question must always bear upon nursing, directly or indirectly.**
**If an answer is required by letter a fee of half-a-crown must be enclosed with the note containing the inquiry.**

*Recent Editions.*

(44) Can you tell me the date of the latest edition of "Recent Materia Medica," by Harwood Lescher (pub. Churchill); and "Materia Medica and Therapeutics," by Mitchell Bruce (pub. Cassell). Also any other reliable modern work on Materia Medica.—*A. S.*

"Recent Materia Medica," 1897. "Materia Medica and Therapeutics," 40th thousand, 1900.

*Cottage Hospital.*

(45) Last year we had fifty in-patients; out of that number twenty were water-works men. I am anxious to know what the latter each cost the hospital—there has been an argument as to what expenses should be included in their cost.—*Lady Superintendent.*

According to the treasurer's statement for last year, the cost of the water-works men was £211, or £10 10s. each.

*The Weir-Mitchell Treatment.*

(46) 1. Whereabouts are the homes or establishmente exclusively devoted to the Rest Cure or Weir-Mitchell treatment?
2. What is the inclusive fee per week ot these homes?
3. Would the treatment be reliable if carried out in an ordinary hospital which advertises as using the system, and would it be less expensive at such hospital, expense being a great consideration in the case?

A number of homes devoted to the Weir-Mitchell treatment are advertised, and their inclusive fees can doubtless be ascertained on application. But it is not safe to rely upon advertisements. The treatment should only be given under the superintendence of a medical man.

*Etiquette.*

(47) Paddie is nursing a mental case to which she was sent by the hospital at which she is being trained. Her three years' novitiate will be ended shortly, and she would be glad to know if afterwards, it would be right for her to continue to attend her present case, as the friends of the patient wish her to.

Cannot "Paddie" ask her matron the same question she has put to us? Then a fair arrangement to both parties could be made.

*Fire.*

(48) Can anyone give me practical advice how to keep a fire up for several hours without attention. It seems impossible in the limited coal space of a London flat to obtain and keep small coal and briquettes?—*Patient.*

Not at all impossible. A cheap packing case with lid holds a considerable quantity of fuel, and stands anywhere. If the fine coal be treated with coal saver, it will keep the fire good with very little attention.

*Lepers.*

(49) Will you kindly give me information about nursing lepers and to whom to apply.—*Evelina.*

There are no lepers to nurse in England: you must therefore apply to institutions abroad for particulars. "Burdett's Hospitals and Charities" gives the more noted leper asylums in the Colonies.

*Nurse Child.*

(50) Will you kindly tell me how to get the care of a lady's child. I have been told one can easily be obtained by writing to the lying-in hospitals, but I want more information?—*S. E. S.*

Your best plan would be to advertise in a medical paper. The lying-in hospitals are fully given in "Burdett's Hospitals and Charities."

*Sewing Maid.*

(51) Will you please tell me how I can get my daughter as under-sewing maid into a hospital or home? She is quite strong, but a little lame. *Nurse W.*

See advertisements and advertise.

*Toronto.*

(52) Would you kindly tell me if, after I finish my three years' training in England, I could get work in a Canadian hospital or home, Toronto preferred; and what salaries are offered?—*Leslie.*

The Lady Superintendent of the Victorian Order of Nurses for Canada, 578 Somerset Street, Ottawa, could possibly help you. Nurses of this order are employed in every province, and receive $300 (that is £60) a year, all found.

*Co-operation.*

(53) Is there a nurse co-operative home in Manchester? If so, please give me the address.—*Edith S.*

There is none managed by a committee worked on co-operative lines.

**Standard Books of Reference.**

"The Nursing Profession: How and Where to Train." 2s. net; post free 2s. 4d.
"The Nurses' Dictionary of Medical Terms." 2s.
"Burdett's Series of Nursing Text-Books." 1s. each.
"A Handbook for Nurses." (Illustrated.) 5s.
"Nursing: Its Theory and Practice." New Edition. 3s. 6d.
"Helps in Sickness and to Health." Fifteenth Thousand. 5s.
"The Physiological Feeding of Infants." 1s.
"The Physiological Nursery Chart." 1s.; post free, 1s. 3d.
"Hospital Expenditure: The Commissariat." 2s. 6d.

All these are published by THE SCIENTIFIC PRESS, Ltd., and may be obtained through any bookseller or direct from the publishers, 28 and 29 Southampton Street, London, W.C.

